# Short-Chain Fatty Acids as Modulators of the Gut–Immune Axis in Inflammatory Bowel Disease: Linking Metabolism, Immune Regulation, and Epithelial Barrier Function

**DOI:** 10.3390/ijms27219710

**Published:** 2026-10-29

**Authors:** Dominika Piątek, Aneta Sokal-Dembowska, Małgorzata Strycharz-Dudziak, Sara Jarmakiewicz-Czaja

**Affiliations:** 1 Chair and Department of Conservative Dentistry with Endodontics, Medical University of Lublin, 20-093 Lublin, Poland; dominika.piatek@umlub.edu.pl (D.P.); malgorzata.strycharz-dudziak@umlub.edu.pl (M.S.-D.); 2 Faculty of Health Sciences and Psychology, Collegium Medicum, University of Rzeszów, 35-959 Rzeszów, Poland; asokal@ur.edu.pl; 3 University Center for Research and Development in Health Sciences, University of Rzeszów, 35-959 Rzeszów, Poland

**Keywords:** short-chain fatty acids, inflammatory bowel disease, epithelial barrier, butyrate, mucus layer, tight junction, propionate, acetate

## Abstract

Inflammatory bowel diseases (IBDs), which include Crohn’s disease (CD) and ulcerative colitis (UC), are chronic conditions associated with abnormalities in the immune response, gut microbiota dysbiosis, and disruption of the intestinal barrier homeostasis. A growing body of evidence suggests that the gut–immune axis plays a key role in the pathogenesis of IBD, and that metabolites of microbial origin influence the regulation of the immune response and the maintenance of intestinal barrier integrity. Particular importance is attributed to short-chain fatty acids (SCFAs), such as acetate, propionate, and butyrate. The aim of this review is to present the current knowledge regarding the role of SCFAs as modulators of the gut–immune axis in IBD, with particular emphasis on their mechanisms of action and therapeutic potential. Physiologically, SCFAs are produced through the fermentation of dietary fiber by the gut microbiota. However, in IBD, the composition of the gut microbiota is often disrupted. SCFAs influence the expression of inflammatory genes and regulate the function of immune cells. Furthermore, butyrate in particular strengthens the integrity of the intestinal barrier by improving the function of tight junctions (TJs), increasing mucus production, and supporting epithelial regeneration. SCFAs represent an important link between diet, the gut microbiota, and immunity. Strategies to increase their availability may represent a promising approach to supporting IBD treatment, but they require further evaluation in well-designed clinical trials involving humans.

## 1. Introduction

Inflammatory bowel disease (IBD) is a group of chronic autoimmune diseases of multifactorial etiology with a remitting–relapsing course, among which two main clinical forms can be distinguished: Crohn’s disease (CD) and ulcerative colitis (UC). CD, characterized by transmural, segmental inflammation, can affect any part of the gastrointestinal tract and can lead to complications such as fibrous damage, fistulas and abscesses, while UC affects the large intestine and involves continuous inflammation of the mucous membrane leading to symptoms such as diarrhea, abdominal pain, and weight loss [1,2]. According to Global Burden of Disease base in 2021 with information gathered from 204 countries and territories, there were 3,830,119 global prevalent cases of IBD. From 1990 to 2021, there was an 88.30% increase in global incident cases of IBD (199236 in 1990 and 375,140 in 2021), 98.07% increase in global deaths associated with IBD (21,418 in 1990 and 42,423 in 2021) and 59.22% increase in DALYs (disability-adjusted life years; from 948861 cases in 1990 to 1,510,784 cases in 2021) [3,4].

The gut–immune axis plays a key role in the pathogenesis of IBD. It mediates interactions between the gut microbiota and immune responses. Disruptions of this axis can trigger inadequate immune responses and apparent changes in the gut’s microbiota contributing to homeostasis disorders and to the development and progression of the disease. The immune system is categorized into two subdivisions: the innate immune system, with the epithelial cell barrier, antimicrobial proteins (AMPs), defensins, and dendritic cells; and the adaptive immune system, with T helper cells TH1, TH2 and TH17, regulatory T cells (Treg cells) and B Lymphocytes (B cells) [5,6]. Bridging between these two systems occurs through cytokines secreted by antigen-presenting cells (APCs), which play a key role in the differentiation and activation of T cells. Among them, we can distinguish: pro-inflammatory cytokines Tumor Necrosis Factor (TNF-α), Interleukin-1 (IL-1), IL-1β, Interleukin-18 (IL-18), and Interleukin 6 (IL-6), and anti-inflammatory cytokine Interleukin 10 (IL-10) [7]. The proper function of both immune system components depends on the precise regulation of immune responses, encompassing the activation and suppression of distinct immune cell subsets, the controlled production of cytokines and chemokines, and the modulation of intracellular signaling pathways. This coordinated regulation is essential for ensuring effective immune responses while preventing excessive inflammation and tissue damage [8].

More than 100 trillion microorganisms, including bacteria, viruses, fungi, and protozoa, constitute the human microbiome, which co-exist to form a complex ecological community. The gastrointestinal (GI) tract represents a major ecological niche, hosting a large and diverse population of these microorganisms [9]. In a healthy microbiome of the GI, the predominant types of bacteria are *Bacillota*, *Actinomycetota*, *Bacteroidota* and *Verrucomicrobiota*, while the distribution of species depends on the section of the GI tract in which they are found. The stomach and small intestine contain relatively small bacteria populations and predominant species are *Streptococcus*, *Prevotella*, and *Veillonella* for high-stress conditions in the stomach, and *Pseudomonadota* and *Bacillota* for a small intestine environment rich in mono- and disaccharides. The large intestine, rich in complex polysaccharides that are indigestible by humans, provides an excellent area for greater number and diversity of colonizing bacteria such as *Bacteroides*, *Bifidobacterium*, and *Eubacteriales*, which have the ability to produce enzymes that break down these compounds [10]. Proper regulatory functioning of the immune system–gut microbiota axis in a newborn leads to the formation of a permanent and homeostatic host/commensal relationship, which is of long-term importance for human health [11]. The gut microbiome affects microbial homeostasis, the production of nutrients necessary for the body’s growth, metabolism and detoxification, but its composition and functional capacity continuously evolve throughout the host’s lifespan to bring mutual benefits [7]. Changes in the microbiota at every stage of life can lead to the disruption of the balance, but in mothers or newborns may predispose to diseases associated with dysregulated barrier responses, such as asthma [11].

The severity of IBD has been shown to be associated with a disorder in the normal microbiota referred to as dysbiosis. In dysbiosis, there is a possibility that under the influence of the altered environment, intestinal commensal bacteria may transform into pathobionts. Studies on animal models have shown that Germ-Free (GF) rodents genetically susceptible to disease will not develop intestinal inflammation, while Specific Pathogen-Free (SPF) mice show symptoms of colitis. Inflammation of the large intestine is also spontaneously developed by mice in which the gene responsible for IL-10, which is a strong anti-inflammatory cytokine, has been eliminated. It has also been noted that a specific gut microbiota can be preventively covered with high levels of IgA, and the difference in microbial coverage can be a tool to distinguish between potentially pathogenic bacteria and commensals [12]. Changes in the microbiota have been observed in patients with IBD, including a decrease in the number of *Bacillota*, an increase in the number of *Pseudomonadota*, and several *Bacteroidota*, leading to impaired immunomodulatory functions and a decrease in the integrity of the intestinal barrier, allowing inflammation to occur [10].

In patients with IBD, altered levels of gut microbiota metabolites are detected compared to healthy individuals. These findings may be important as bacterial metabolites such as short-chain fatty acids (SCFAs), secondary bile acids (BAs), and indole and its derivatives have a number of functions supporting the host organism [10,13]. Recent studies show the important role of SCFAs, which are indicated by scientists as being responsible for regulating the structure of the gut microbiota, strengthening the intestinal epithelial barrier with significant impact on intestinal homeostasis, energy metabolism and immune response modulation, and serving as crucial mediators of host–microbial interactions [13,14].

SCFAs are monocarboxylic (saturated fatty) acids containing up to six carbon atoms, produced by intestinal bacteria from undigested carbohydrates and resistant starch via the fermentation process [13,14]. The most important of these are acetate, butyrate and propionate, which represent 90–95% of the SCFAs, with the molar ratio in the human colon and feces approximately 60:20:20, respectively [13,15]. These proportions can change depending on factors like diet, age and diseases, which can have an impact on the ratio of commensal intestinal bacteria [16]. The average daily production of SCFAs in the human colon is 500–600 mmol [15].

This review focuses on the molecular mechanisms linking the metabolism of short-chain fatty acids (SCFAs) to immune regulation and the function of the intestinal epithelial barrier. Particular attention is given to SCFA-mediated signaling pathways, their effects on immune cells, and the latest evidence regarding dysbiosis and impaired SCFA production in IBD. This comprehensive perspective aims to provide a more detailed understanding of the interactions between microbial metabolism, immune responses, and epithelial barrier integrity in IBD.

## 2. Short-Chain Fatty Acids: Origin and Metabolism

Several groups of gut bacteria are responsible for the production of SCFAs. Depending on the metabolite produced, different genera play a main role. Acetate is mainly synthesized by *Akkermansia*, *Bifidobacterium*, *Bacteroides*, *Prevotella*, *Ruminococcus*, *Clostridium*, and *Streptococcus*. Butryrate originates from *Coprococcus eutactus*, *C. comes*, *Anaerostipes*, *Agathobacter rectalis*, *C. catus*, *Anaerobutyricum hallii*, *Roseburia*, and *Faecalibacterium prausnitzii*, but members of the *Bacillota* phylum are considered the most efficient producers of butyrate. Propionate formation is mainly due to *Bacteroides*, *Dialister*, *Veillonella*, *Salmonella*, *Megasphaera elsdenii*, *Pseudocoprococcus catus*, *Roseburia inulinivorans*, *Phascolarctobacterium succinatutens*, and *Blautia obeum* activity [2]. Some bacteria, by expressing genes encoding enzymes involved in butyrate synthesis, allow other bacteria such as *Pseudomonadota* and *Fusobacteriota*, which are not usually associated with significant SCFA production, to synthesize this compound [14]. The substrates for SCFA production are usually substances indigestible to the host, such as microbially accessible carbohydrates, including plant-derived inulin, polysaccharides, and resistant starch [17]. Gut microbiota can also metabolize endogenous substrates such as exfoliated epithelial cells, mucus and intestinal secretions, the utilization of which may have a potential impact on the SCFA profile [18]. The type and amount of SCFAs depends on the microbiome present (its type and abundance), the type and availability of substrate, the rate of intestinal transit, and the pH level, which influences the composition of the gut microbiota and enzyme activity [2,14]. The type of SCFA is related to gut microbiota composition and may change throughout life depending on factors such as age and diet. In the early stages of life, *Bifidobacteria* predominate, metabolizing human milk oligosaccharides to produce mainly acetate. After cessation of breastfeeding, there is an increase in the number of *Bacillota*, which break down complex carbohydrates and produce butyrate and other SCFAs [2].

### 2.1. Microbial Production of SCFAs

Gut microbiota ferment nondigestible carbohydrates under anaerobic conditions, hydrolyzing them into oligosaccharides and monosaccharides. These are metabolized via the Embden–Meyerhof–Parnas pathway (for hexoses) or the pentose phosphate pathway (for pentoses), generating phosphoenolpyruvate (PEP), which is further converted into pyruvate. During glycolysis, NADH is produced and must be reoxidized to maintain cellular redox balance [19]. 

Three main mechanisms facilitate the disposal of excess reducing equivalents. First, pyruvate is reduced to lactate or ethanol, regenerating NAD^+^. Second, primary fermenters produce molecular hydrogen (H_2_) through ferredoxin-dependent hydrogenases, either via an exergonic pathway involving pyruvate:ferredoxin oxidoreductase or an endergonic pathway involving NADH:ferredoxin oxidoreductase. The latter requires low H_2_ partial pressure, maintained by hydrogen-consuming microorganisms. Third, some bacteria use an anaerobic electron transport chain in which PEP is carboxylated to oxaloacetate and subsequently reduced to fumarate. Electrons from NADH are transferred to fumarate via NADH dehydrogenase and fumarate reductase, generating a proton gradient that drives ATP synthesis. The resulting succinate can be converted into propionate [19].

The major end products of carbohydrate fermentation are short-chain fatty acids (SCFAs), primarily acetate, propionate, and butyrate. Acetate is produced from acetyl-CoA through hydrolysis or via the Wood–Ljungdahl pathway, which incorporates CO_2_ into acetyl-CoA [20]. Propionate is synthesized mainly through the succinate pathway, involving fumarate reduction, or through the acrylate pathway, in which lactate is converted to propionate. Both pathways contribute to NADH reoxidation. It also can be synthesized via the propanediol pathway from deoxyhexose sugars (fucose or rhamnose). Butyrate is formed by the condensation of two acetyl-CoA molecules, followed by reduction to butyryl-CoA. The final conversion to butyrate occurs either through the classical phosphotransbutyrylase–butyrate kinase pathway, which generates ATP, or via butyryl-CoA:acetate CoA-transferase, which uses extracellular acetate and produces butyrate and acetyl-CoA. The latter pathway appears to predominate in the human gut and reflects metabolic cross-feeding between acetate-producing and butyrate-producing bacteria. Lactate-metabolizing bacteria can synthesize butyrate via the conversion of lactate to acetyl-CoA [21].

SCFA production depends on metabolic interactions within the microbial community and on host–microbiota symbiosis. The consumption of H_2_ by other microorganisms prevents its accumulation, which would otherwise inhibit the oxidation of NADH and reduce fermentation efficiency. Host-derived CO_2_ and bicarbonate also support microbial metabolism, particularly fumarate-dependent pathways. Furthermore, bicarbonate secreted into the intestinal lumen can neutralize protons generated during SCFA production, contributing to the regulation of intestinal pH [15,18]. A summary of the metabolic pathways involved in microbial SCFA production is presented in Table 1.

### 2.2. SCFA Absorption, Distribution, Receptors and Signaling Pathways

SCFAs are rapidly and efficiently absorbed in the colon, with approximately 90–95% of the produced metabolites entering the colonic epithelium and less than 10% being excreted in the feces [22]. They represent the primary energy source for colonocytes and enterocytes, providing approximately 60–70% of their energy requirements. Their uptake across the apical membrane of colonocytes occurs through multiple complementary mechanisms, including passive diffusion of the protonated form, SCFA/HCO_3_^−^ exchange, and carrier-mediated transport. The latter is primarily facilitated by monocarboxylate transporter 1 (MCT1; SLC16A1) and sodium-coupled monocarboxylate transporter 1 (SMCT1; SLC5A8), which exhibit distinct substrate affinities and transport kinetics. Among the major SCFAs, SMCT1 displays the highest affinity for butyrate, whereas acetate and propionate are transported less efficiently [23,24]. SCFAs are oxidized by colonocytes to meet their high metabolic demands, with butyrate representing the principal oxidative substrate. After absorption, butyrate undergoes mitochondrial β-oxidation followed by metabolism through the tricarboxylic acid (TCA) cycle (Krebs cycle), resulting in efficient ATP generation. This process supplies a substantial proportion of the energy required for epithelial maintenance and supports intestinal barrier function and cellular metabolism [25,26]. Following absorption, SCFAs enter the portal circulation and undergo differential metabolic processing. Propionate is transported predominantly to the liver, where it serves as an important substrate for gluconeogenesis and other metabolic pathways. Acetate undergoes comparatively limited hepatic extraction and therefore represents the predominant SCFA in the systemic circulation, enabling its distribution to peripheral tissues, including skeletal muscle, adipose tissue, the heart, and the brain. Owing to extensive first-pass hepatic metabolism, circulating concentrations of butyrate and propionate remain substantially lower than those of acetate [14,15,27,28].

Table 2 presents molecular targets, such as HDAC (histone deacetylase), Toll-like receptors, GPCRs (G protein-coupled receptors), NLRP3 (nod-like receptor pyrin domain) inflammasomes, and MCT1 and MCT4, and mechanisms involved in SCFA-mediated host–microbiota interactions and immune regulation [2,13,14,15,16,17,29,30,31,32,33,34].

## 3. Dysbiosis and SCFA Deficiency in IBD

The considerable interindividual variability of the human gut microbiome has precluded the establishment of a universal taxonomic profile that defines a “healthy” microbiota. Current evidence suggests that health is better characterized by the functional capacity, ecological stability, and resilience of the microbial community rather than by the presence or abundance of specific bacterial taxa. Accordingly, a healthy gut microbiota is regarded as a dynamic ecosystem that maintains host homeostasis through its involvement in nutrient metabolism, maintenance of intestinal barrier integrity, and regulation of both innate and adaptive immune responses [35].

An appropriately structured and functionally competent commensal microbiota also provides protection against pathogen colonization through the phenomenon of colonization resistance. This protective mechanism encompasses competition for available nutrients and ecological niches on the intestinal mucosal surface, thereby limiting pathogen establishment, as originally described by Stecher and Hardt [36]. In addition, SCFAs reduce luminal pH and modulate the intestinal microenvironment, thereby inhibiting the growth and persistence of many enteric pathogens [37].

Intestinal dysbiosis is considered one of the central biological features of IBD, although its precise role in disease initiation remains unresolved. Rather than representing a single causative factor, dysbiosis is currently viewed as a dynamic disturbance of the intestinal microbial ecosystem that interacts with host genetics, environmental exposures, epithelial barrier integrity, and immune responses. Increasing evidence indicates that disease development results from the disruption of the reciprocal relationship between the host and the gut microbiota rather than from the presence or absence of individual bacterial species. Consequently, current research has shifted from describing taxonomic changes toward understanding functional alterations within the microbial community and their impact on intestinal homeostasis [38].

### 3.1. Gut Microbiota Alterations

One of the most consistent findings in IBD is a reduction in gut microbial diversity. However, decreased α-diversity is not disease-specific, as it is also observed in other chronic inflammatory and metabolic disorders, and is influenced by disease activity, treatment, diet, and methodological factors. Therefore, reduced diversity is considered a marker of ecological instability rather than a specific diagnostic feature [39].

Despite substantial interindividual variability, several reproducible taxonomic alterations have been described. IBD is characterized by depletion of beneficial obligate anaerobes, particularly members of the *Ruminococcaceae* and *Lachnospiraceae* families, including *Faecalibacterium prausnitzii* and *Roseburia* spp., which are important producers of butyrate. In contrast, facultative anaerobic *Pseudomonadota*, including adherent-invasive *Escherichia coli* (AIEC), are frequently enriched. Current evidence suggests that AIEC acts as an opportunistic pathobiont whose expansion is promoted by the inflammatory intestinal environment rather than serving as a primary cause of disease [10,15,35,40,41].

Table 3 presents an overview of the latest studies related to the identification and abundance of intestinal bacteria determined from human stool collected in studies of IBD patients compared to a healthy control group [42,43,44,45,46,47,48].

### 3.2. Reduced SCFA Production

Increasing evidence indicates that functional alterations of the microbiota are more relevant to IBD pathogenesis than taxonomic composition alone. Multi-omics studies have demonstrated that distinct microbial communities may share similar metabolic disturbances, supporting the concept of functional dysbiosis. A hallmark of this process is reduced production of short-chain fatty acids, particularly butyrate, which impairs epithelial metabolism, barrier integrity, and immune regulation while promoting the expansion of facultative anaerobic bacteria. Altered metabolism of bile acids, tryptophan, and sphingolipids further disrupts host–microbiota signaling pathways involved in mucosal homeostasis [49].

Reduced production of SCFAs is one of the major functional consequences of gut dysbiosis in IBD. The most pronounced decrease concerns butyrate, although reduced concentrations of acetate and propionate have also been reported, particularly during active disease. The decline in SCFA production is primarily associated with the depletion of obligate anaerobic bacteria belonging to the families *Ruminococcaceae* and *Lachnospiraceae*, including *Faecalibacterium prausnitzii*, *Roseburia* spp., *Agathobacter rectalis*, and *Anaerostipes* spp., which are among the principal butyrate-producing microorganisms in the healthy colon. In addition, dysbiosis disrupts microbial cross-feeding networks, whereby acetate produced by species such as *Bifidobacterium* spp. is utilized by butyrate-producing bacteria [15,40,50]. Consequently, the loss of these metabolic interactions further limits butyrate synthesis. Reduced abundance of propionate-producing bacteria, including members of the genus *Bacteroides* and *Veillonella*, may also contribute to lower propionate levels. Decreased SCFA availability impairs colonocyte energy metabolism, compromises epithelial barrier integrity, and alters mucosal immune regulation by limiting regulatory T-cell differentiation and anti-inflammatory signaling. These metabolic disturbances are therefore considered a key mechanism linking gut dysbiosis with persistent intestinal inflammation in IBD [49,51].

### 3.3. Clinical Correlations

Current evidence supports a bidirectional relationship between dysbiosis and inflammation. Rather than representing a purely causal event, dysbiosis both contributes to and is reinforced by intestinal inflammation, creating a self-sustaining cycle that promotes disease progression. Nevertheless, substantial heterogeneity among microbiome studies and the marked interpersonal variability of healthy microbiota continue to limit the identification of robust microbial biomarkers for routine clinical use [38,52]. Lower abundance of butyrate-producing bacteria, particularly *Faecalibacterium prausnitzii* and *Roseburia* spp., as well as reduced fecal butyrate concentrations, has been associated with increased mucosal inflammation and impaired epithelial healing [53]. Moreover, alterations in SCFA metabolism have been linked to reduced responsiveness to therapy and persistent intestinal inflammation, suggesting that microbial metabolic dysfunction may serve as a clinically relevant indicator of disease progression. Although these associations are well documented, current evidence does not support the use of dysbiosis or SCFA levels as independent diagnostic or prognostic biomarkers due to substantial interindividual variability and methodological heterogeneity among studies [54].

## 4. SCFAs as Modulators of Immune Responses

### 4.1. Innate Immunity

Innate (non-specific) immunity constitutes the body’s first line of defense. The response to pathogen invasion is rapid but is not directed against a single pathogen, which makes it non-specific [55,56]. Innate immunity relies on epithelial cells and immune system cells, including macrophages, dendritic cells, NK cells, and others [57].

One of the most frequently reported effects of SCFAs is the regulation of macrophage function. In a study by Luhrs et al., following the topical administration of butyrate to patients with UC (ulcerative colitis), the researchers observed inhibition of NF-κB activation in macrophages [58]. Consequently, this may lead to a reduction in pro-inflammatory cytokines and, thus, a reduction in inflammation [29]. Additionally, Liang et al. indicate that butyrate promotes the polarization of macrophages from the M1 (inflammatory) phenotype to the M2 (anti-inflammatory) phenotype, which, through communication with goblet cells, influences mucus production. As a result, the integrity of the intestinal barrier is strengthened, and epithelial regenerative processes are enhanced [59]. Experimental studies in a NASH model indicate that butyrate may reduce the pro-inflammatory response of macrophages and promote an M2-like phenotype by inhibiting HDAC. These findings provide additional mechanistic evidence supporting the immunomodulatory properties of butyrate [60]. 

Experimental studies involving LPS-stimulated dendritic cells have shown that butyrate inhibits the activation and maturation of dendritic cells, in part by regulating cellular metabolism [61]. DCs have high levels of membrane MHC expression, which enables them to activate T cells. In turn, butyrate reduces the expression of surface molecules, thereby promoting a decrease in the ability of DCs to stimulate T cells [62]. Additionally, the production of anti-inflammatory mediators, including IL-10, increases, which helps maintain anti-inflammatory response [63].

The effect of SCFAs has also been demonstrated in neutrophils. G protein-coupled receptors (GPRs) are the receptors that detect SCFAs. The GPR43 receptor is present, among other places, on the surface of neutrophils, and upon activation by SCFAs, it is responsible, among other things, for regulating the chemotaxis of these cells toward the site of inflammation [64]. It has also been shown that butyrate, acetate, and propionate can influence the phagocytic capacity of neutrophils [65]. Additionally, Aoyama et al. indicate that propionate and butyrate can induce neutrophil apoptosis through mechanisms dependent on HDAC inhibition, which may limit the excessive activation of these cells during inflammation [66].

A growing number of studies also point to the role of SCFAs in regulating NLRP3 inflammasome activity. Wang et al. indicate that, under inflammatory conditions, SCFAs, particularly butyrate and propionate, can activate the NLRP3 inflammasome in macrophages, leading to the release of IL-1β without inducing classical pyroptosis. This mechanism depends, among other factors, on HDAC inhibition, loss of IL-10 expression, and caspase-8 activation. This suggests that the effects of SCFAs are highly context-dependent and can be both anti-inflammatory and pro-inflammatory [67]. Yuan et al. reached similar conclusions, observing that SCFAs exert a complex effect on inflammasome activity, the outcome of which depends on cell type and the local inflammatory environment [33]. In contrast, in another in vivo study, Shi et al. found that propionate administration inhibited NLRP3 inflammasome activation in UC induced by sodium dextran sulfate (DSS) [68]. Additional evidence from experimental models of spinal cord injury suggests that butyrate may inhibit NLRP3 inflammasome activation through mechanisms involving HDAC1 [69].

### 4.2. Adaptive Immunity

The adaptive immune system is responsible for the specific immune response and the development of immune memory, which enables the effective protection of the organism against re-exposure to the same pathogen. The main cells involved in this response are T cells and B cells [70].

SCFAs, particularly butyrate, support T-cell function by enhancing their effector activity and promoting the formation of long-lived CD8+ T memory cells. These effects result from the reprogramming of cellular metabolism and the regulation of gene expression, which translates into a stronger immune response [71,72]. SCFAs influence cytokine expression and T-cell activity by inhibiting HDACs and regulating acetyl-CoA-dependent epigenetic processes. Their beneficial effects have been demonstrated in numerous inflammatory diseases [73]. SCFAs regulate T-cell differentiation, leading to a reduction in the Th17 response and IL-17 production, while simultaneously modulating the function of regulatory T cells [74]. Evidence summarized in reviews of rheumatoid arthritis suggests that SCFAs may modulate the Th17/Treg balance through metabolic, epigenetic, and cytokine-mediated mechanisms [75,76]. SCFAs influence changes in the gut microbiota, which in turn affect the Th1/Th2 balance, thereby modulating the balance within the immune system [77]. Chen et al. observed that the administration of sodium butyrate may reduce the expression of IL-6 and TNF-α in animal models of induced colitis [78].

Butyrate and propionate also modulate B-cell function by inhibiting the expression of the chemokine receptor CCR10 in activated cells, which may limit their migration and participation in the immune response [79]. However, in a human study by Gill et al., the authors demonstrated that increased exposure to SCFAs through a 21-day dietary intervention had a subtle effect on circulating B cells, leading to a slight reduction in the number of total and naive B cells. Nevertheless, the authors note that further research in this area is necessary [80]. Butyrate also supports the functions of regulatory B cells (Bregs) by enhancing their immunosuppressive activity through activation of the aryl hydrocarbon receptor (AhR) [81].

SCFAs play a fundamental role in the regulation of adaptive immunity.

## 5. SCFAs and the Intestinal Barrier

The intestinal barrier is a fundamental protective element of the organism, separating the internal environment from the lumen of the gastrointestinal tract. It consists of several layers. The outer layer is composed of the commensal gut microbiota, a mucus layer, secretory immunoglobulin A (sIgA), and antimicrobial peptides (AMPs). The epithelial barrier consists of intestinal epithelial cells and tight junction (TJ) proteins. In addition to the chemical and epithelial barriers, there is also an immunological barrier, which consists of immune cells, and a vascular barrier, which is formed by endothelial cells also connected by TJs [82]. Intestinal barrier dysfunction is one of the key elements of IBD pathogenesis, leading to increased intestinal epithelial permeability [83]. Impaired intestinal barrier integrity in IBD involves increased epithelial permeability, TJ dysfunction, changes in the mucus layer, and abnormal communication between epithelial cells, the gut microbiota, and the immune system [84].

### 5.1. Mucus Layer

The mucus layer in the intestines is essential for the proper functioning of the intestinal barrier. The functioning of the intestinal mucus layer results from complex interactions between host glycoproteins and the gut microbiota. A growing body of evidence suggests that appropriately selected nutrients and commensal bacteria can support the integrity of the intestinal mucosal barrier [85]. Furthermore, the selective permeation of nutrients provides an environment for secretory immunoglobulin A (sIgA) and antimicrobial peptides [86]. Intestinal mucus, secreted mainly by goblet cells, consists primarily of mucin (MUC2) and, in the large intestine, forms two layers: an inner layer that is virtually free of bacteria under physiological conditions, and an outer layer colonized by commensal microorganisms [87,88]. One of the main mechanisms of action of butyrate is the inhibition of HDAC [89]. When butyrate is the primary energy source for colonocytes, MUC2 expression increases significantly. However, the effect of this metabolite is related to the type of mucin, and a stronger association has been demonstrated for MUC5B and MUC3. No such associations have been observed for acetate and propionate [90]. Gaudier et al. indicate that butyrate may influence the properties of the mucosal barrier by regulating mucin glycosylation, and the strongest effect of butyrate was an increase in the expression of galectin-1 (LGALS1) [91]. In their study on the mechanisms of butyrate’s protective effects in UC, Liang et al. demonstrated that this metabolite supports the restoration of the intestinal mucosal barrier by inducing M2 macrophage polarization, which consequently leads to an increase in the number of goblet cells and enhanced expression of mucin 2 (MUC2) [59]. In turn, Wang et al. emphasized that dysfunction of, among others, goblet cells is one of the key elements in the pathogenesis of CD, contributing to damage to the mucosal barrier. The authors suggest that butyrate, among other compounds, could offer a promising approach to supplement primary therapy in this patient population [92]. In addition to stimulating MUC2 synthesis, butyrate also influences the maturation and differentiation of goblet cells. Butyrate-dependent signaling via the GPR109A receptor affects the number of specialized goblet cells between the crypts [93]. The effect of SCFAs on protective factors in the mucosal barrier has also been demonstrated. SCFAs may enhance the activity of IgA-producing B cells by providing metabolic substrates. Additionally, the authors noted that SCFA levels in feces were associated with sIgA levels, although this relationship was complex [94]. SCFAs play a key role in maintaining the normal structure and function of the intestinal mucus layer. By stimulating MUC2 gene expression, promoting goblet cell differentiation, and increasing the production of antimicrobial substances, they strengthen the mucosal barrier.

### 5.2. Epithelial Cells

Butyrate provides energy to colon cells, supporting their metabolism and helping to maintain proper intestinal barrier function. Butyrate is transported into cells via monocarboxylate transporter 1 (MCT1) and sodium-coupled monocarboxylate transporter 1 (SMCT1) [95]. SCFAs, especially butyrate, modulate the function of intestinal epithelial cells (IECs) by inducing the expression of antimicrobial peptides, such as β-defensins. This effect is GPR43-dependent and involves the activation of signaling pathways such as mTOR and STAT3, which contribute to the maintenance of intestinal homeostasis [96]. Furthermore, butyrate, by inhibiting HDACs and downregulating NF-κB pathways, may limit IEC activation by inflammatory stimuli and reduce the production of pro-inflammatory mediators [97,98]. Activation of the FFAR2/GPR43 and FFAR3/GPR41 receptors by SCFAs may also indirectly lead to a reduction in the production of pro-inflammatory cytokines [95]. Intestinal stem cells (ISCs), located at the base of the intestinal crypts, are a key component of the intestinal epithelium; due to their ability to proliferate and differentiate, they enable continuous regeneration of the epithelium and its complete renewal, which occurs on average every 3–5 days. Farhadipour et al. investigated the effects of SCFAs on ISCs. They observed that SCFAs derived from fermented dietary fiber by the gut microbiota can significantly alter ISC differentiation by shifting it toward absorptive enterocytes at the expense of secretory cells. By inhibiting HDACs, SCFAs activate the Notch pathway, which promotes changes in ISC differentiation [99]. Through activation of the GPR41/GPR43 receptors and the extracellular signal-regulated kinase (ERK), mTOR, and STAT3 pathways, SCFAs promote crypt cell proliferation, migration, and IEC turnover [100]. Bilotta et al. observed that propionate stimulates the migration of intestinal epithelial cells in vivo and in vitro, thereby accelerating wound healing [101]. In turn, Grouls et al. conducted a study on the effect of SCFAs on IECs derived from human induced pluripotent stem cells (iPSCs). The authors indicate that each SCFA elicited a distinct transcriptional response; the effect was dose-dependent, but the strongest effect was observed with butyrate, a moderate effect with propionate, and the weakest effects with acetate [102]. In summary, SCFAs play a key role in the regeneration of the intestinal epithelium. By providing energy to intestinal epithelial cells, regulating the expression of genes responsible for cell proliferation and differentiation, and supporting the migration of epithelial cells to the site of damage, they contribute to maintaining the integrity of the mucosal barrier.

### 5.3. Tight Junctions

Tight junctions (TJs), located in the most apical part of the intestinal epithelium, act as a barrier, regulating intercellular permeability and maintaining the polarization of epithelial cells. TJs consist of claudins and occludin, which are transmembrane proteins linked by the zona occludens (ZO-1, ZO-2, ZO-3). Permeability across tight junctions (TJs) occurs primarily via two pathways: the selective pore pathway, regulated mainly by claudins, and the less selective leak pathway, regulated by multiple factors, including occludin [103]. In an in vitro study using cell cultures, Saleri et al. demonstrated that propionate and acetate had a beneficial effect on cell viability, and this effect was associated with increased expression of claudin-4 (CLDN4). Furthermore, butyrate and lactate strengthened the structure of tight junctions (ZO-1, occludin (OCLN)). All SCFAs tested reduced the expression of TNF-α and NF-κB. However, the authors note that all these effects of SCFAs were dose-dependent [104]. Another study also demonstrated that SCFAs have the ability to increase TJ expression and improve ZO-1 organization in the cell membrane, and that they protect against LPS-induced barrier damage by, among other things, limiting TJ structural disruption. The researchers also note that SCFAs inhibit NLRP3 inflammasome activation [105]. Andrani et al. reached similar conclusions, observing that acetate and propionate reduced nitric oxide (NO) production following lipopolysaccharide (LPS) stimulation, limited NF-κB activation, and increased TJ protein expression [106]. Additionally, butyrate may block the IL-13-induced decrease in trans-epithelial electrical resistance (TEER) and increase in claudin-2 expression [107]. TJ dysfunction resulting, among other things, from impaired regulation by non-coding RNAs (miRNAs) may lead to increased intestinal permeability [108]. In their study, Metzler-Zebeli et al. indicate that SCFAs may act as early as the prenatal period, influencing, among other things, TJP expression, the regulation of SCFA receptors, and signaling pathways [109]. In summary, experimental evidence suggests that SCFAs, particularly butyrate, may support intestinal barrier integrity.

## 6. Therapeutic Perspectives of SCFA-Mediated Pathways

Diet is one of the most important environmental factors regulating the composition and activity of the gut microbiota, and thus the levels of metabolites it produces, including SCFAs. The most physiological way to increase the availability of SCFAs is to provide fermentation substrates for gut bacteria in the form of dietary fiber [110]. Harris et al. indicate that the quality of SCFAs produced is influenced by diet type, food preparation methods, intestinal transit time, and the composition of the gut microbiota [111]. In their systematic review, Sobh et al. report that an increase in SCFAs is observed following resistant starch supplementation [112]. Consuming foods rich in resistant starch—such as chilled potatoes, green bananas, legume seeds, and certain whole-grain products—may promote the production of butyrate in particular [113,114]. In addition, butyrate is produced primarily following the consumption of inulin. In contrast, rhamnose, arabinose, and guar gum may increase the proportion of propionate, while galactose, lactose, and lactulose promote increased acetate production [111]. SCFA production also depends on the composition of the gut microbiota and the metabolic interactions between individual bacterial species. Acetate is produced mainly by *Bifidobacterium* spp., propionate by *Bacteroidota* (e.g., *Prevotella* spp.), *Phascolarctobacterium succinatutens*, *Veillonella* spp., and *Blautia* spp., and butyrate is largely produced by *Faecalibacterium*, *Roseburia*, *Eubacterium*, and *Anaerostipes* [53,115]. Reduced availability of fermentation substrates and disturbances in the composition of the gut microbiota can lead to decreased SCFA production, which is observed, among others, in patients with IBD [116]. A diet rich in fruits, vegetables, nuts, legumes, and whole grains promotes greater diversity in the gut microbiota and increases SCFA production. An example of such a dietary pattern is the Mediterranean diet [117]. In contrast, a Western-style diet, rich in ultra-processed foods containing saturated fats and simple carbohydrates, leads to dysbiosis, a reduction in the abundance of SCFA-producing bacteria, and a weakening of the intestinal barrier integrity [118,119].

Increasing SCFA concentrations through dietary modification is not always possible, particularly in patients with IBD. However, significantly lower concentrations of butyrate are observed in these patient groups, especially during periods of disease activity [38]. For this reason, direct SCFA supplementation is attracting increasing interest. One of the most commonly used forms of supplementation is sodium butyrate. This form is characterized by good chemical stability and the ability to deliver butyrate ions to the gastrointestinal tract. In an animal model of IBD, sodium butyrate was shown to alleviate disease symptoms and improve intestinal barrier integrity by increasing the expression of ZO-1 and occludin. Additionally, the authors point to the inhibition of macrophage ferroptosis following supplementation with the compound [120]. In another study, the authors also report changes in the composition of the gut microbiota, improved intestinal histology, and reduced inflammation following supplementation [121]. Similar results were also obtained by Chen et al., who observed a decrease in the expression of pro-inflammatory cytokines and an increase in the expression of tight junctions [78]. In human studies involving sodium butyrate supplementation, beneficial changes in the composition of the gut microbiota were demonstrated [122]. One of the derivatives of butyrate is tributyrin. This compound is characterized by greater stability and better bioavailability compared to free butyrate. Ye et al. investigated the effects of tributyrin in an animal model of induced inflammatory diarrhea. They found that supplementation with this compound can increase the expression of SCFA transporters and the number of goblet cells, which contributes to maintaining the homeostasis of the intestinal barrier [123]. Tributyrin improved the clinical condition of mice following antibiotic therapy. Additionally, an increase in gut microbiota diversity and a decrease in the expression of pro-inflammatory cytokines were observed [124]. Tributyrin reduces the severity of intestinal inflammation in rats; furthermore, it was observed to inhibit the morphological changes characteristic of pyroptosis, which plays a key role in the pathogenesis of IBD [125].

## 7. Conclusions

SCFAs are key mediators of the gut–immune axis, linking gut microbiota activity, regulation of the immune response, and intestinal barrier homeostasis. In IBD, a reduction in the number of SCFA-producing bacteria and impaired SCFA production contribute to the exacerbation of inflammation and dysfunction of the intestinal barrier. Strategies aimed at increasing SCFA availability, including dietary modifications and interventions targeting the gut microbiota, may have therapeutic potential in IBD. However, clinical evidence remains limited and inconsistent, highlighting the need for well-designed clinical trials to establish their efficacy and potential role in personalized IBD management.

## Figures and Tables

**Table 1:** Metabolic pathways of microbial SCFA production.

SCFAs	Metabolic Pathway	Simplified Scheme
Acetate	Acetogenesis	Pyruvate → Acetyl-CoA → Acetate
	Wood–Ljungdahl pathway	CO_2_ → Formate → Methyl-CoFeSP → Acetyl-CoA → Acetate ↓ CO → Acetyl-CoA → Acetate
Propionate	Acrylate pathway	Pyruvate → [Lactate → Lactoyl-CoA] → Propionate
	Succinate pathway	PEP → Oxaloacetate → [Malate → Fumarate → Succinate → Methylmalonyl-CoA] → Propionate
	Propanediol pathway	Fucose or Rhamnose → [Propionaldehyde → Propionyl-CoA] → Propionate
Butyrate	Butyrate kinase pathway	Pyruvate → [Acetyl-CoA → Acetoacetyl-CoA] → Butyrate
	Synthesis from lactate	Lactate → Butyrate
	Synthesis from acetate	Acetate → Butyrate

**Table 2:** Molecular targets and interaction effects of SCFAs in IBD.

Molecular Targets		Location	Type of SCFA Interacting with the Target	Interaction Effect	Role in IBD	References
***Enzymes***						
**HDAC (histone deacetylase)**		Cell nucleus	Butyrate	Inhibition of HDAC	Upregulation of anti-inflammatory gene expression Promotion of Treg differentiation → Secretion of cytokines by Tregs (IL-10) → Suppression of inflammation	[11,15]
***Receptors***						
**GPCRs** **(G protein-coupled receptors**)	**GPR41 (known also as FFAR3)**	Digestive tract epithelial cells, adipocytes, skeletal muscle and liver	Propionate and butyrate mostly, then acetate	Induction of PYY and GLP1 secretion by L cells; impact on multiple tissues (cardiovascular system, pancreas, brain)	Regulation of macrophage activity	[2,10,12,13,14,17]
	**GPR43 (known also as FFAR2)**	Digestive tract epithelial cells, adipocytes, skeletal muscle and liver	At similar rate acetate, propionate, and butyrate, but propionate remains the most important activator	Recognition by enteroendocrine L cells during expression in immune cells; increase in Treg cells and IL-10 (anti-inflammatory function)	Promotion of IgA production, suppression of intestinal inflammation Increase in Amphiregulin expression in dendritic cells, facilitating tissue repair Inhibition of nuclear factor kappa-B activity	
	**GPR109A (known also as HCAR2)**	Intestinal tract, partial immune cells, brain, prostate, spleen and adipocytes	Butyrate	IL-10 production resulting in anti-inflammatory properties	Inhibition of IL-23 production	
**Toll-like receptors**		Transmembrane receptors of immune cells	Butyrate (mostly), acetate, propionate	Limitation of excessive activation of NF-κB and production of pro-inflammatory cytokines	Contribution to intestinal immune homeostasis	[18,19]
***Downstream Signaling Complex***						
**NLRP3 (nod-like receptor pyrin domain) inflammasomes**		Macrophages, endothelial cells	Butyrate, acetate, propionate	Activation of caspase-1 mediating the release of IL1β	Recent studies suggest that activation of NLRP3 inhibits development of IBD (via SCFAs binding to GPR43 and GPR109A)	[14,20]
***Transporters***						
**MCT1 and MCT4**		Colonocytes	Butyrate, acetate, propionate	Mediation of SCFA absorption into host cells	Promotion of cellular metabolism	[21]

HDAC, histone deacetylase; GPCRs, G protein-coupled receptors; GPR41, G protein-coupled receptor 41; GPR43, G protein-coupled receptor 43; GPR109A, G protein-coupled receptor 109A; FFAR3, free fatty acid receptor 3; FFAR2, free fatty acid receptor 2; NLRP3, nod-like receptor pyrin domain 3; MCT1, monocarboxylate transporter 1; MCT4, monocarboxylate transporter 4; PYY, peptide tyrosine tyrosine; GLP1, Glucagon-like peptide-1; NF-κB, nuclear factor kappa-light-chain-enhancer of activated B cells; IL1β, Interleukin-1 beta; IL-10, Interleukin-10; IL-23, Interleukin-23; IgA, immunoglobulin A; IBD, inflammatory bowel disease; HCAR2, Hydroxycarboxylic Acid Receptor 2.

**Table 3:** Gut microbiota alterations in inflammatory bowel diseases.

Reference; Year of Publication	Country and Year of the Study (if Mentioned)	Studied Groups	Sample Origin	Method	Bacteria	Results (Bacterial Abundance in Relation to Healthy Control)
[42]; 2025	Spain	12 CD, 13 UC and 5 HC	Stool samples	Metagenomics	*Collinsella*, *Dialister*, *Fusobacterium*, *Parabacteroides*, *Ruminococcus*, *Succinivibrio*, *Prevotella*	↑ CD (active phase)
					*Bacteroides*, *Anaerostipes*, *Mediterraneibacter torques*, *Erysipelotrichaceae*, *Lachnoclostridium*, *Prevotellaceae*	↓ CD (active phase) ↓ UC
					*Christensenellaceae*, *Coprococcus*, *Prevotella*, *Faecalibacterium*, *Collinsella*, *Streptococcus*	↑ UC
[43]; 2025	2021–2022 Korea	41 IBD vs 11 HC	Stool samples	16S rRNA gene amplicon sequencing and culturomics	*Pseudomonadota*	↑
					*Actinomycetota*	↓
					*Enterobacteriaceae*	↑
[44]; 2025	2017–2019 Spain	103 CD, 144 UC, and 49 HC	Stool samples	Shotgun metagenomics	*Escherichia coli*	↑
					*Faecalibacterium prausnitzii*	↓
					*Adlercreutzia genus*	↓
					*Shigella genus*	↑
[45]; 2025	2022–2023 Iran	13 CD, 17 UC and 30 HC	Stool samples	Real-time qPCR	*Bacillota*	↑
					*Lactobacillus*	↓
					*Bifidobacterium* spp.	↓
					*Fusobacterium* spp.	↑
					*Bacteroides fragilis*	↑
					*Faecalibacterium prausnitzii*	↓
[46]; 2025	Three-year study Sweden	64 CD, 55 UC and 134 HC	Stool samples	Shotgun metagenomics	*Coprococcus eutactus*	↓ CD
					*Clostridium* sp. *CAG 167*	↓ CD
					*Faecalibacterium prausnitzii*	↓ UC
					*Akkermansia muciniphila*	↓ UC
[47]; 2020	2014–2018 Kazan, Russian Federation	41 CD, 43 UC and 42 HC	Stool samples	16S rRNA sequencing and shotgun metagenomics	Bacillota (*Faecalibacterium*, *Eubacterium*, *Ruminococcus*, and *Roseburia*)	↓
					*Lactobacillales* (*Lactobacillus* and *Streptococcus*)	↑ UC
					*Bacteroidota*	↓
					*Butyricimonas* and *Odoribacter*	↓
					*Akkermansia*	↓
					*Pseudomonadota and Actinomycetota* (*Escherichia*, *Haemophilus*, *Bifidobacterium*, and *Collinsella*)	↑
[48]; 2021	Cork, Ireland, and Manitoba, Canada	303 CD, 228 UC and 161 HC	Stool samples	16S rRNA gene sequencing	*Mediterraneibacter gnavus*	↑ CD
					*Eggerthella lenta*	↑ CD
					*Lachnospira eligens and Agathobacter rectalis*	↓ CD
					*Faecalibacterium prausnitzii*	↓ CD
					*E. lenta*	↑ UC
					*Holdemania filiformis*	↑ UC
					*Clostridium innocuum*	↑ UC
					*Lachnospira eligens*	↓ UC
					*Clostridium aminobutyricum*	↓ UC

CD, Crohn’s disease; UC, ulcerative colitis; HC, healthy control.

## Data Availability

No new data were created or analyzed in this study. Data sharing is not applicable to this article.

## References

[1] Silaghi A., Constantin V.D., Socea B., Banu P., Sandu V., Andronache L.F., Dumitriu A.S., Paunica S. (2022). Inflammatory Bowel Disease: Pathogenesis, Diagnosis and Current Therapeutic Approach. J. Mind Med. Sci..

[2] Ottria R., Mirmajidi S., Ciuffreda P. (2026). Gut Microbiota-Derived Short-Chain Fatty Acids in Inflammatory Bowel Disease: Mechanistic Insights into Gut Inflammation, Barrier Function, and Therapeutic Potential. Int. J. Mol. Sci..

[3] Lin D., Jin Y., Shao X., Xu Y., Ma G., Jiang Y., Xu Y., Jiang Y., Hu D. (2024). Global, regional, and national burden of inflammatory bowel disease, 1990–2021: Insights from the global burden of disease 2021. Int. J. Colorectal Dis..

[4] Ruan G., Sun Y., Yu Z., Bai X., Yang H., Qian J. (2025). Global, regional, and national burden of inflammatory bowel disease from 1990 to 2021: Findings from the Global Burden of Disease 2021. Gastroenterol. Rep..

[5] Bretto E., Urpì-Ferreruela M., Casanova G.R., González-Suárez B. (2025). The Role of Gut Microbiota in Gastrointestinal Immune Homeostasis and Inflammation: Implications for Inflammatory Bowel Disease. Biomedicines.

[6] Geremia A., Biancheri P., Allan P., Corazza G.R., Di Sabatino A. (2014). Innate and adaptive immunity in inflammatory bowel disease. Autoimmun. Rev..

[7] Almusallam D., Alhabardi S. (2025). A systematic review on the dynamic interplay between the immune system and gut microbiome in inflammatory bowel disease patients. Future J. Pharm. Sci..

[8] Choy M.C., Visvanathan K., De Cruz P. (2017). An Overview of the Innate and Adaptive Immune System in Inflammatory Bowel Disease. Inflamm. Bowel Dis..

[9] Murphy J.F. (2018). The human microbiome: An emerging paradigm for better health. MOJ Immunol..

[10] White Z., Cabrera I., Kapustka I., Sano T. (2023). Microbiota as key factors in inflammatory bowel disease. Front. Microbiol..

[11] Belkaid Y., Hand T.W. (2014). Role of the Microbiota in Immunity and Inflammation. Cell.

[12] Li Y. (2026). Gut microbiota in the pathogenesis and treatment of inflammatory bowel disease: A critical review of mechanisms and therapeutic advances. Front. Med..

[13] Zhang D., Jian Y.-P., Zhang Y.-N., Li Y., Gu L.-T., Sun H.-H., Liu M.-D., Zhou H.-L., Wang Y.-S., Xu Z.-X. (2023). Short-chain fatty acids in diseases. Cell Commun. Signal..

[14] Shin Y., Han S., Kwon J., Ju S., Choi T.G., Kang I., Kim S.S. (2023). Roles of Short-Chain Fatty Acids in Inflammatory Bowel Disease. Nutrients.

[15] Anachad O., Taouil A., Taha W., Bennis F., Chegdani F. (2023). The Implication of Short-Chain Fatty Acids in Obesity and Diabetes. Microbiol. Insights.

[16] Ratajczak W., Rył A., Mizerski A., Walczakiewicz K., Sipak O., Laszczyńska M. (2019). Immunomodulatory potential of gut microbiome-derived short-chain fatty acids (SCFAs). Acta Biochim. Pol..

[17] Zhang Z., Zhang H., Chen T., Shi L., Wang D., Tang D. (2022). Regulatory role of short-chain fatty acids in inflammatory bowel disease. Cell Commun. Signal..

[18] Facchin S., Bertin L., Bonazzi E., Lorenzon G., De Barba C., Barberio B., Zingone F., Maniero D., Scarpa M., Ruffolo C. (2024). Short-Chain Fatty Acids and Human Health: From Metabolic Pathways to Current Therapeutic Implications. Life.

[19] Den Besten G., van Eunen K., Groen A.K., Venema K., Reijngoud D.-J., Bakker B.M. (2013). The role of short-chain fatty acids in the interplay between diet, gut microbiota, and host energy metabolism. J. Lipid Res..

[20] Ragsdale S.W., Pierce E. (2008). Acetogenesis and the Wood–Ljungdahl pathway of CO2 fixation. Biochim. et Biophys. Acta (BBA) Proteins Proteom..

[21] Duncan S.H., Holtrop G., Lobley G.E., Calder A.G., Stewart C.S., Flint H.J. (2004). Contribution of acetate to butyrate formation by human faecal bacteria. Br. J. Nutr..

[22] Venegas D.P., De la Fuente M.K., Landskron G., González M.J., Quera R., Dijkstra G., Harmsen H.J.M., Faber K.N., Hermoso M.A. (2019). Short Chain Fatty Acids (SCFAs)-Mediated Gut Epithelial and Immune Regulation and Its Relevance for Inflammatory Bowel Diseases. Front. Immunol..

[23] Sankarganesh P., Bhunia A., Kumar A.G., Babu A.S., Gopukumar S., Lokesh E. (2025). Short-chain fatty acids (SCFAs) in gut health: Implications for drug metabolism and therapeutics. Med. Microecol..

[24] Liu S.H., Yang X.F., Liang L., Bin Song B., Song X.M., Yang Y.J., Alhoot M.A. (2025). Regulatory mechanisms of the gut microbiota-short chain fatty acids signaling axis in slow transit constipation and progress in multi-target interventions. Front. Microbiol..

[25] Nogal A., Valdes A.M., Menni C. (2021). The role of short-chain fatty acids in the interplay between gut microbiota and diet in cardio-metabolic health. Gut Microbes.

[26] Du Y., He C., An Y., Huang Y., Zhang H., Fu W., Wang M., Shan Z., Xie J., Yang Y. (2024). The Role of Short Chain Fatty Acids in Inflammation and Body Health. Int. J. Mol. Sci..

[27] Zhao Y., Chen J., Qin Y., Yuan J., Yu Z., Ma R., Liu F., Zhao J. (2025). Linking Short-Chain Fatty Acids to Systemic Homeostasis: Mechanisms, Therapeutic Potential, and Future Directions. J. Nutr. Metab..

[28] Overby H.B., Ferguson J.F. (2021). Gut Microbiota-Derived Short-Chain Fatty Acids Facilitate Microbiota:Host Cross talk and Modulate Obesity and Hypertension. Curr. Hypertens. Rep..

[29] Zhao H., Zhou Y., Xu J., Zhang Y., Wang H., Zhao C., Huang H., Yang J., Huang C., Li Y. (2024). Short-chain fatty acid-producing bacterial strains attenuate experimental ulcerative colitis by promoting M2 macrophage polarization via JAK/STAT3/FOXO3 axis inactivation. J. Transl. Med..

[30] Lin M.Y., de Zoete M.R., van Putten J.P.M., Strijbis K. (2015). Redirection of Epithelial Immune Responses by Short-Chain Fatty Acids through Inhibition of Histone Deacetylases. Front. Immunol..

[31] Sam Q.H., Ling H., Yew W.S., Tan Z., Ravikumar S., Chang M.W., Chai L.Y.A. (2021). The Divergent Immunomodulatory Effects of Short Chain Fatty Acids and Medium Chain Fatty Acids. Int. J. Mol. Sci..

[32] Luke A.M., N S.K., Hegde M.N., Hegde N.N. (2025). Metabolic interplay of SCFA’s in the gut and oral microbiome: A link to health and disease. Front. Oral Health.

[33] Yuan X., Wang L., Bhat O.M., Lohner H., Li P.-L. (2018). Differential effects of short chain fatty acids on endothelial Nlrp3 inflammasome activation and neointima formation: Antioxidant action of butyrate. Redox Biol..

[34] Akhtar M., Chen Y., Ma Z., Zhang X., Shi D., Khan J.A., Liu H. (2021). Gut microbiota-derived short chain fatty acids are potential mediators in gut inflammation. Anim. Nutr..

[35] Foppa C., Rizkala T., Repici A., Hassan C., Spinelli A. (2023). Microbiota and IBD: Current knowledge and future perspectives. Dig. Liver Dis..

[36] Stecher B., Hardt W.-D. (2011). Mechanisms controlling pathogen colonization of the gut. Curr. Opin. Microbiol..

[37] Krishnamurthy H.K., Pereira M., Bosco J., George J., Jayaraman V., Krishna K., Wang T., Bei K., Rajasekaran J.J. (2023). Gut commensals and their metabolites in health and disease. Front. Microbiol..

[38] Chulenbayeva L., Jarmukhanov Z., Kaliyekova K., Kozhakhmetov S., Kushugulova A. (2025). Quantitative Alterations in Short-Chain Fatty Acids in Inflammatory Bowel Disease: A Systematic Review and Meta-Analysis. Biomolecules.

[39] Vestergaard M.V., Allin K.H., Eriksen C., Zakerska-Banaszak O., Arasaradnam R.P., Alam M.T., Kristiansen K., Brix S., Jess T. (2023). Gut microbiota signatures in inflammatory bowel disease. United Eur. Gastroenterol. J..

[40] Khorsand B., Aghdaei H.A., Nazemalhosseini-Mojarad E., Nadalian B., Nadalian B., Houri H. (2022). Overrepresentation of Enterobacteriaceae and Escherichia coli is the major gut microbiome signature in Crohn’s disease and ulcerative colitis; a comprehensive metagenomic analysis of IBDMDB datasets. Front. Cell. Infect. Microbiol..

[41] Kaur S., Thakur R. (2025). Gut microbiota and inflammatory bowel disease: Mechanisms, clinical implications, and future directions. Mol. Biol. Rep..

[42] Arribas-Rodríguez E., García-Pizarro M., De Prado Á., Santiago-Carretero M., Hernández M., Jiménez B.V., García-Alonso J., Barrio J., Eiros J.M., Arranz E. (2025). Fungal-Bacterial Dysbiosis in IBD: Microbial Biomarkers of Disease Activity. Microbiologyopen.

[43] Park H., Yeo S., Lee T., Han Y., Ryu C.B., Huh C.S. (2025). Culture-based characterization of gut microbiota in inflammatory bowel disease. Front. Microbiol..

[44] Orejudo M., Gómez M.J., Riestra S., Rivero M., Gutiérrez A., Rodríguez-Lago I., Fernández-Salazar L., Ceballos D., Benítez J.M., Aguas M. (2025). Exploration of fecal microbiota in newly diagnosed patients with inflammatory bowel disease using shotgun metagenomics. Front. Cell. Infect. Microbiol..

[45] Ahmadi A., Kouhsari E., Razavi S., Mohamadzadeh N., Besharat S., Vakili M.A., Amiriani T. (2025). Comparative analysis of dominant gut microbiota in Inflammatory Bowel Disease patients and healthy individuals: A case-control study. New Microbes New Infect..

[46] Prast-Nielsen S., Granström A.L., Kiasat A., Ahlström G., Edfeldt G., Rautiainen S., Boulund F., Andersson F.O., Lindberg J., Schuppe-Koistinen I. (2025). Associations of the intestinal microbiota with plasma bile acids and inflammation markers in Crohn’s disease and ulcerative colitis. Sci. Rep..

[47] Sasso G.L., Khachatryan L., Kondylis A., Battey J.N.D., Sierro N., A Danilova N., Grigoryeva T.V., I Markelova M., Khusnutdinova D.R., Laikov A.V. (2020). Inflammatory Bowel Disease–Associated Changes in the Gut: Focus on Kazan Patients. Inflamm. Bowel Dis..

[48] Clooney A.G., Eckenberger J., Laserna-Mendieta E., A Sexton K., Bernstein M.T., Vagianos K., Sargent M., Ryan F.J., Moran C., Sheehan D. (2021). Ranking microbiome variance in inflammatory bowel disease: A large longitudinal intercontinental study. Gut.

[49] Deleu S., Machiels K., Raes J., Verbeke K., Vermeire S. (2021). Short chain fatty acids and its producing organisms: An overlooked therapy for IBD?. EBioMedicine.

[50] Lloyd-Price J., Arze C., Ananthakrishnan A.N., Schirmer M., Avila-Pacheco J., Poon T.W., Andrews E., Ajami N.J., Bonham K.S., Brislawn C.J. (2019). Multi-omics of the gut microbial ecosystem in inflammatory bowel diseases. Nature.

[51] Louis P., Flint H.J. (2017). Formation of propionate and butyrate by the human colonic microbiota. Environ. Microbiol..

[52] Stange E.F. (2024). Dysbiosis in inflammatory bowel diseases: Egg, not chicken. Front. Med..

[53] Fusco W., Lorenzo M.B., Cintoni M., Porcari S., Rinninella E., Kaitsas F., Lener E., Mele M.C., Gasbarrini A., Collado M.C. (2023). Short-Chain Fatty-Acid-Producing Bacteria: Key Components of the Human Gut Microbiota. Nutrients.

[54] Ryan F.J., Ahern A.M., Fitzgerald R.S., Laserna-Mendieta E.J., Power E.M., Clooney A.G., O’donoghue K.W., McMurdie P.J., Iwai S., Crits-Christoph A. (2020). Colonic microbiota is associated with inflammation and host epigenomic alterations in inflammatory bowel disease. Nat. Commun..

[55] Kaur B.P., Secord E. (2021). Innate Immunity. Immunol. Allergy Clin. N. Am..

[56] Pradeu T., Thomma B.P., Girardin S.E., Lemaitre B. (2024). The conceptual foundations of innate immunity: Taking stock 30 years later. Immunity.

[57] Hoffmann J., Akira S. (2013). Innate immunity. Curr. Opin. Immunol..

[58] Lührs H., Gerke T., Müller J.G., Melcher R., Schauber J., Boxberger F., Scheppach W., Menzel T. (2002). Butyrate Inhibits NF-κB Activation in Lamina Propria Macrophages of Patients with Ulcerative Colitis. Scand. J. Gastroenterol..

[59] Liang L., Liu L., Zhou W., Yang C., Mai G., Li H., Chen Y. (2022). Gut microbiota-derived butyrate regulates gut mucus barrier repair by activating the macrophage/WNT/ERK signaling pathway. Clin. Sci..

[60] Sarkar A., Mitra P., Lahiri A., Das T., Sarkar J., Paul S., Chakrabarti P. (2023). Butyrate limits inflammatory macrophage niche in NASH. Cell Death Dis..

[61] Wu L., Luo Z., Chen Y., Yan Z., Fu J., Jiang Y., Xu J., Liu Y. (2023). Butyrate Inhibits Dendritic Cell Activation and Alleviates Periodontitis. J. Dent. Res..

[62] Liu L., Li L., Min J., Wang J., Wu H., Zeng Y., Chen S., Chu Z. (2012). Butyrate interferes with the differentiation and function of human monocyte-derived dendritic cells. Cell. Immunol..

[63] Duan H., Wang L., Huangfu M., Li H. (2023). The impact of microbiota-derived short-chain fatty acids on macrophage activities in disease: Mechanisms and therapeutic potentials. Biomed. Pharmacother..

[64] Hayashi A., Nagao-Kitamoto H., Kitamoto S., Kim C.H., Kamada N. (2021). The Butyrate-Producing Bacterium *Clostridium butyricum* Suppresses *Clostridioides difficile* Infection via Neutrophil- and Antimicrobial Cytokine–Dependent but GPR43/109a-Independent Mechanisms. J. Immunol..

[65] Dang A.T., Begka C., Pattaroni C., Caley L.R., Floto R.A., Peckham D.G., Marsland B.J. (2023). Butyrate regulates neutrophil homeostasis and impairs early antimicrobial activity in the lung. Mucosal Immunol..

[66] Aoyama M., Kotani J., Usami M. (2010). Butyrate and propionate induced activated or non-activated neutrophil apoptosis via HDAC inhibitor activity but without activating GPR-41/GPR-43 pathways. Nutrition.

[67] Wang W., Dernst A., Martin B., Lorenzi L., Cadefau-Fabregat M., Phulphagar K., Wagener A., Budden C., Stair N., Wagner T. (2024). Butyrate and propionate are microbial danger signals that activate the NLRP3 inflammasome in human macrophages upon TLR stimulation. Cell Rep..

[68] Shi Y., Xin D., Zhang H., Wang S., Yang M., Liu C. (2025). Propionate alleviates ulcerative colitis by modulating the PI3K/AKT signaling pathway and suppressing NLRP3 inflammasome activation. Food Funct..

[69] Ganggang K., Cheng G., Gang L., Wenwu Z., Di Z., Yan L., Baoshu X., Yiqin W. (2025). From Molecular Docking to Rat Models: Butyrate From a High-Fiber Diet Inhibits HDAC1 and NLRP3 Inflammasome to Alleviate Oxidative Stress and Inflammation After Spinal Cord Injury. J. Neurochem..

[70] Sun L., Wang X., Saredy J., Yuan Z., Yang X., Wang H. (2020). Innate-adaptive immunity interplay and redox regulation in immune response. Redox Biol..

[71] Luu M., Riester Z., Baldrich A., Reichardt N., Yuille S., Busetti A., Klein M., Wempe A., Leister H., Raifer H. (2021). Microbial short-chain fatty acids modulate CD8+ T cell responses and improve adoptive immunotherapy for cancer. Nat. Commun..

[72] Bachem A., Makhlouf C., Binger K.J., de Souza D.P., Tull D., Hochheiser K., Whitney P.G., Fernandez-Ruiz D., Dähling S., Kastenmüller W. (2019). Microbiota-Derived Short-Chain Fatty Acids Promote the Memory Potential of Antigen-Activated CD8^+^ T Cells. Immunity.

[73] Luu M., Visekruna A. (2019). Short-chain fatty acids: Bacterial messengers modulating the immunometabolism of T cells. Eur. J. Immunol..

[74] Li N., Han X., Ruan M., Huang F., Yang L., Xu T., Wang H., Wu H., Shi S., Wang Y. (2024). Prebiotic inulin controls Th17 cells mediated central nervous system autoimmunity through modulating the gut microbiota and short chain fatty acids. Gut Microbes.

[75] Pang A., Pu S., Pan Y., Huang N., Li D. (2025). Short-chain fatty acids from gut microbiota restore Th17/Treg balance in rheumatoid arthritis: Mechanisms and therapeutic potential. J. Transl. Autoimmun..

[76] Lv J., Hao P., Zhou Y., Liu T., Wang L., Song C., Wang Z., Liu Z., Liu Y. (2025). Role of the intestinal flora-immunity axis in the pathogenesis of rheumatoid arthritis-mechanisms regulating short-chain fatty acids and Th17/Treg homeostasis. Mol. Biol. Rep..

[77] Yan Q., Wang W., Fan Z., Wei Y., Yu R., Pan T., Wang N., Lu W., Li B., Fang Z. (2025). Chickpea-resistant starch exhibits bioactive function for alleviating atopic dermatitis via regulating butyrate production. Int. J. Biol. Macromol..

[78] Chen G., Ran X., Li B., Li Y., He D., Huang B., Fu S., Liu J., Wang W. (2018). Sodium Butyrate Inhibits Inflammation and Maintains Epithelium Barrier Integrity in a TNBS-induced Inflammatory Bowel Disease Mice Model. EBioMedicine.

[79] Porbahaie M., Hummel A., Saouadogo H., Coelho R., Savelkoul H., Teodorowicz M., van Neerven R. (2023). Short-chain fatty acids inhibit the activation of T lymphocytes and myeloid cells and induce innate immune tolerance. Benef. Microbes.

[80] A Gill P., Muir J.G., Gibson P.R., van Zelm M.C. (2022). A randomized dietary intervention to increase colonic and peripheral blood SCFAs modulates the blood B- and T-cell compartments in healthy humans. Am. J. Clin. Nutr..

[81] Rosser E.C., Piper C.J., Matei D.E., Blair P.A., Rendeiro A.F., Orford M., Alber D.G., Krausgruber T., Catalan D., Klein N. (2020). Microbiota-Derived Metabolites Suppress Arthritis by Amplifying Aryl-Hydrocarbon Receptor Activation in Regulatory B Cells. Cell Metab..

[82] Sun N., Liu W., Li J., Zhang D., Liu X., Liu J., Hu H. (2025). Composition, function, and detection methods of the intestinal barrier. J. Nutr. Biochem..

[83] Selvakumar B., Samsudin R. (2025). Intestinal Barrier Dysfunction in Inflammatory Bowel Disease: Pathophysiology to Precision Therapeutics. Inflamm. Bowel Dis..

[84] Schoultz I., Keita Å.V. (2019). Cellular and Molecular Therapeutic Targets in Inflammatory Bowel Disease—Focusing on Intestinal Barrier Function. Cells.

[85] Paone P., Cani P.D. (2020). Mucus barrier, mucins and gut microbiota: The expected slimy partners?. Gut.

[86] Martínez-Augustin O., Tena-Garitaonaindia M., Ceacero-Heras D., Jiménez-Ortas Á., Enguix-Huete J.J., Álvarez-Mercado A.I., Ruiz-Henares G., Aranda C.J., Gámez-Belmonte R., de Medina F.S. (2025). Macronutrients as Regulators of Intestinal Epithelial Permeability: Where Do We Stand?. Compr. Rev. Food Sci. Food Saf..

[87] Desai M.S., Seekatz A.M., Koropatkin N.M., Kamada N., Hickey C.A., Wolter M., Pudlo N.A., Kitamoto S., Terrapon N., Muller A. (2016). A Dietary Fiber-Deprived Gut Microbiota Degrades the Colonic Mucus Barrier and Enhances Pathogen Susceptibility. Cell.

[88] Floor E., Su J., Chatterjee M., Kuipers E.S., Ijssennagger N., Heidari F., Giordano L., Wubbolts R.W., Mihăilă S.M., Stapels D.A.C. (2024). Development of a Caco-2-based intestinal mucosal model to study intestinal barrier properties and bacteria–mucus interactions. Gut Microbes.

[89] Della Ragione F., Criniti V., Della Pietra V., Borriello A., Oliva A., Indaco S., Yamamoto T., Zappia V. (2001). Genes modulated by histone acetylation as new effectors of butyrate activity. FEBS Lett..

[90] Gaudier E., Jarry A., Blottière H.M., de Coppet P., Buisine M.P., Aubert J.P., Laboisse C., Cherbut C., Hoebler C. (2004). Butyrate specifically modulates *MUC* gene expression in intestinal epithelial goblet cells deprived of glucose. Am. J. Physiol.-Gastrointest. Liver Physiol..

[91] Gaudier E., Forestier L., Gouyer V., Huet G., Julien R., Hoebler C. (2004). Butyrate regulation of glycosylation-related gene expression: Evidence for galectin-1 upregulation in human intestinal epithelial goblet cells. Biochem. Biophys. Res. Commun..

[92] Wang Z., Shen J. (2024). The role of goblet cells in Crohn’ s disease. Cell Biosci..

[93] Nirello V.D., Araújo N., de Assis H.C., Moreno-Gonzalez M., Ruiz P., Castro P.R., Shealy N.G., Shelton C., Fernandes M.F., de Oliveira S. (2025). Microbiota shape the colon epithelium controlling inter-crypt absorptive goblet cells via butyrate–GP R109A signalling. Gut Microbes.

[94] Davias A., Verghese M., Bridgman S.L., Tun H.M., Field C.J., Hicks M., Pei J., Hicks A., Moraes T.J., Simons E. (2025). Gut microbiota metabolites, secretory immunoglobulin A and Bayley-III cognitive scores in children from the CHILD Cohort Study. Brain Behav. Immun. Health.

[95] Fu Y., Lyu J., Wang S. (2023). The role of intestinal microbes on intestinal barrier function and host immunity from a metabolite perspective. Front. Immunol..

[96] Zhao Y., Chen F., Wu W., Sun M., Bilotta A.J., Yao S., Xiao Y., Huang X., Eaves-Pyles T.D., Golovko G. (2018). GPR43 mediates microbiota metabolite SCFA regulation of antimicrobial peptide expression in intestinal epithelial cells via activation of mTOR and STAT3. Mucosal Immunol..

[97] Fawad J.A., Luzader D.H., Hanson G.F., Moutinho T.J., McKinney C.A., Mitchell P.G., Brown-Steinke K., Kumar A., Park M., Lee S. (2022). Histone Deacetylase Inhibition by Gut Microbe-Generated Short-Chain Fatty Acids Entrains Intestinal Epithelial Circadian Rhythms. Gastroenterology.

[98] Peng K., Xiao S., Xia S., Li C., Yu H., Yu Q. (2024). Butyrate Inhibits the HDAC8/NF-κB Pathway to Enhance Slc26a3 Expression and Improve the Intestinal Epithelial Barrier to Relieve Colitis. J. Agric. Food Chem..

[99] Farhadipour M., Arnauts K., Clarysse M., Thijs T., Liszt K., Van der Schueren B., Ceulemans L.J., Deleus E., Lannoo M., Ferrante M. (2023). SCFAs switch stem cell fate through HDAC inhibition to improve barrier integrity in 3D intestinal organoids from patients with obesity. iScience.

[100] Park J.-H., Kotani T., Konno T., Setiawan J., Kitamura Y., Imada S., Usui Y., Hatano N., Shinohara M., Saito Y. (2016). Promotion of intestinal epithelial cell turnover by commensal bacteria: Role of short-chain fatty acids. PLoS ONE.

[101] Bilotta A.J., Ma C., Yang W., Yu Y., Yu Y., Zhao X., Zhou Z., Yao S., Dann S.M., Cong Y. (2021). Propionate Enhances Cell Speed and Persistence to Promote Intestinal Epithelial Turnover and Repair. Cell. Mol. Gastroenterol. Hepatol..

[102] Grouls M., Janssen A.W.F., Duivenvoorde L.P.M., Hooiveld G.J.E.J., Bouwmeester H., van der Zande M. (2022). Differential gene expression in iPSC-derived human intestinal epithelial cell layers following exposure to two concentrations of butyrate, propionate and acetate. Sci. Rep..

[103] Arumugam P., Saha K., Nighot P. (2024). Intestinal Epithelial Tight Junction Barrier Regulation by Novel Pathways. Inflamm. Bowel Dis..

[104] Saleri R., Borghetti P., Ravanetti F., Cavalli V., Ferrari L., De Angelis E., Andrani M., Martelli P. (2022). Effects of different short-chain fatty acids (SCFA) on gene expression of proteins involved in barrier function in IPEC-J2. Porc. Health Manag..

[105] Feng Y., Wang Y., Wang P., Huang Y., Wang F. (2018). Short-Chain Fatty Acids Manifest Stimulative and Protective Effects on Intestinal Barrier Function Through the Inhibition of NLRP3 Inflammasome and Autophagy. Cell. Physiol. Biochem..

[106] Andrani M., Borghetti P., Ravanetti F., Cavalli V., Ferrari L., De Angelis E., Martelli P., Saleri R. (2023). Acetate and propionate effects in response to LPS in a porcine intestinal co-culture model. Porc. Health Manag..

[107] Huang X., Oshima T., Tomita T., Fukui H., Miwa H. (2021). Butyrate Alleviates Cytokine-Induced Barrier Dysfunction by Modifying Claudin-2 Levels. Biology.

[108] Zhao X., Zeng H., Lei L., Tong X., Yang L., Yang Y., Li S., Zhou Y., Luo L., Huang J. (2021). Tight junctions and their regulation by non-coding RNAs. Int. J. Biol. Sci..

[109] Metzler-Zebeli B.U., Koger S., Sharma S., Sener-Aydemir A., Ruczizka U., Kreutzmann H., Ladinig A. (2022). Short-Chain Fatty Acids Modulate Permeability, Motility and Gene Expression in the Porcine Fetal Jejunum Ex Vivo. Nutrients.

[110] Bai Y., Zhou X., Zhao J., Wang Z., Ye H., Pi Y., Che D., Han D., Zhang S., Wang J. (2022). Sources of Dietary Fiber Affect the SCFA Production and Absorption in the Hindgut of Growing Pigs. Front. Nutr..

[111] Harris H.C., Morrison D.J., Edwards C.A. (2020). Impact of the source of fermentable carbohydrate on SCFA production by human gut microbiota *in vitro*—A systematic scoping review and secondary analysis. Crit. Rev. Food Sci. Nutr..

[112] Sobh M., Montroy J., Daham Z., Sibbald S., Lalu M., Stintzi A., Mack D., A Fergusson D. (2022). Tolerability and SCFA production after resistant starch supplementation in humans: A systematic review of randomized controlled studies. Am. J. Clin. Nutr..

[113] Knudsen K.E.B., Lærke H.N., Hedemann M.S., Nielsen T.S., Ingerslev A.K., Nielsen D.S.G., Theil P.K., Purup S., Hald S., Schioldan A.G. (2018). Impact of Diet-Modulated Butyrate Production on Intestinal Barrier Function and Inflammation. Nutrients.

[114] Raigond P., Ezekiel R., Raigond B. (2014). Resistant starch in food: A review. J. Sci. Food Agric..

[115] Singh V., Lee G., Son H., Koh H., Kim E.S., Unno T., Shin J.-H. (2023). Butyrate producers, “The Sentinel of Gut”: Their intestinal significance with and beyond butyrate, and prospective use as microbial therapeutics. Front. Microbiol..

[116] Li G., Lin J., Zhang C., Gao H., Lu H., Gao X., Zhu R., Li Z., Li M., Liu Z. (2021). Microbiota metabolite butyrate constrains neutrophil functions and ameliorates mucosal inflammation in inflammatory bowel disease. Gut Microbes.

[117] Seethaler B., Nguyen N.K., Basrai M., Kiechle M., Walter J., Delzenne N.M., Bischoff S.C. (2022). Short-chain fatty acids are key mediators of the favorable effects of the Mediterranean diet on intestinal barrier integrity: Data from the randomized controlled LIBRE trial. Am. J. Clin. Nutr..

[118] Martini D., Godos J., Bonaccio M., Vitaglione P., Grosso G. (2021). Ultra-Processed Foods and Nutritional Dietary Profile: A Meta-Analysis of Nationally Representative Samples. Nutrients.

[119] Atzeni A., Hernández-Cacho A., Khoury N., Babio N., Belzer C., Vioque J., Corella D., Fitó M., Clish C., Vidal J. (2025). The link between ultra-processed food consumption, fecal microbiota, and metabolomic profiles in older mediterranean adults at high cardiovascular risk. Nutr. J..

[120] Chen N., Luo S., Zhou X., Zhu B., Liu Y., He H., Luo S., Sun S. (2026). Sodium Butyrate Alleviates IBD by Modulating SIRT1-Involved Ferroptosis and Inhibition of Macrophage Ferroptosis. Nutrients.

[121] Liu Y., Chen N., He H., Liu L., Sun S. (2025). Sodium butyrate alleviates DSS-induced inflammatory bowel disease by inhibiting ferroptosis and modulating ERK/STAT3 signaling and intestinal flora. Ann. Med..

[122] Facchin S., Vitulo N., Calgaro M., Buda A., Romualdi C., Pohl D., Perini B., Lorenzon G., Marinelli C., D’iNcà R. (2020). Microbiota changes induced by microencapsulated sodium butyrate in patients with inflammatory bowel disease. Neurogastroenterol. Motil..

[123] Ye Z., Kini A., Tan Q., Woltemate S., Vital M., Nikolovska K., Seidler U. (2025). Oral tributyrin treatment affects short-chain fatty acid transport, mucosal health, and microbiome in a mouse model of inflammatory diarrhea. J. Nutr. Biochem..

[124] Yang N., Lan T., Han Y., Zhao H., Wang C., Xu Z., Chen Z., Tao M., Li H., Song Y. (2023). Tributyrin alleviates gut microbiota dysbiosis to repair intestinal damage in antibiotic-treated mice. PLoS ONE.

[125] Xu X., Huang Z., Huang Z., Lv X., Jiang D., Huang Z., Han B., Lin G., Liu G., Li S. (2024). Butyrate attenuates intestinal inflammation in Crohn’s disease by suppressing pyroptosis of intestinal epithelial cells via the cGSA-STING-NLRP3 axis. Int. Immunopharmacol..

